# Lifestyle behaviours can mediate socio-economic and sex inequalities in children’s oral health: findings from Children’s Dental Health Survey 2013, England, Wales, and Northern Ireland

**DOI:** 10.1007/s40368-026-01166-2

**Published:** 2026-01-20

**Authors:** K. Zhou, A. Najwa Mohd Khairuddin, J. E. Gallagher, M. Ide, J. Kang

**Affiliations:** 1 Faculty of Dentistry, Oral, and Craniofacial Sciences, King’s College London, London, United Kingdom; 2 Department of Community Oral Health & Clinical Prevention, Faculty of Dentistry, University of Malaya, Kuala Lumpur, Malaysia

**Keywords:** Mediation analysis, Oral health inequalities, Paediatric preventive dentistry, Oral health behaviours

## Abstract

**Background:**

Oral health inequalities in adults are well documented but understudied in children.

**Aim:**

To investigate oral health inequality amongst children within the United Kingdom (UK) by sex and socio-economic status (SES), and whether such inequality may be mediated by healthy lifestyle.

**Design:**

Secondary analysis of the Children's Dental Health Survey 2013 data for England, Wales, and Northern Ireland, including questionnaire responses on demographics, lifestyle, and clinically examined oral health measures in children aged 5, 8, 12, and 15 years in the UK. Descriptive statistics on the oral health inequalities and mediation analyses were performed, assessing lifestyle factors (regular dental attendance, sugary drink intake, and toothbrushing frequency) that potentially mediate sex and SES inequalities in oral health.

**Results:**

A total of 9,866 children were included in this study. Oral health inequalities were observed by sex and SES amongst all aspects of oral health (dental caries, oral health-related quality of life, self-reported oral health, gingival status, and basic periodontal examination). Compared with males (35.8%), fewer females (24.1%, *p* < 0.001) had self-reported good dental health. Children from lower SES groups had more dental caries experience, i.e., decayed, missing, and filled teeth (DMFT), than those from middle and higher SES groups (1.0 ± 2.0 in lower vs. 0.8 ± 1.9 in middle vs. 0.4 ± 1.3 in higher, *p* = 0.027). Furthermore, there was evidence of lifestyle factors mediating the associations between sex, SES, and adolescents’ oral health at 12 and 15 years.

**Conclusion:**

Inequality was observed for children’s oral health by sex and SES; however, regular dental attendance, having less sugary drink intake and optimal toothbrushing mediated these associations in adolescents.

## Introduction

Oral diseases are the most common health issue in the world, with 3.5 billion cases of untreated oral diseases globally in 2017, significantly affecting daily life and well-being through pain, eating difficulties, and communication problems (Bernabe et al. 2020). Oral health is associated with general health. Amongst adults, poor oral health has been associated with several systemic symptoms, such as stroke, respiratory infections, poor diabetic control, and an increased risk of dementia, potentially due to the spread of oral bacteria and/or the chronic inflammatory response associated with oral infection (Li et al. 2000; Larvin et al. 2021, 2023; Gao et al. 2024). Health starts from conception through early childhood (2019). Amongst children, poor oral health has a huge impact on nutrition intake, mental health, and speech development (Peres et al. 2019; Watt et al. 2024), and children with poor oral health are more likely to experience dental pain, leading to school absences and subsequently poor academic performance (Petersen et al. 2005). Poor oral health in childhood is a predictor of disease in later life (Sheiham and Sabbah 2010).

With increasing recognition of the importance of oral health, previous studies have investigated risk factors associated with oral health inequality, such as socio-economic status (SES) and sex (Porter et al. 2016). For the impact of SES on oral health inequalities, individuals with lower SES, both adults and children, are more likely to have worse oral health than their advantaged peers (Public Health England 2021). Children in deprived families show poorer oral hygiene and periodontal health and higher caries experience (Rouxel and Chandola 2018), and more likely to end up admitted to hospital for tooth extractions (Firman et al. 2024; Kaddour et al. 2023). However, when it comes to the association between sex and oral health, research amongst adults showed that females exhibited higher dental caries experience (Lukacs 2011), whilst males were more prone to periodontal disease (Shiau and Reynolds 2010). However, in relation to children, several studies on dental caries showed inconsistent results: some studies have revealed sex inequalities in dental caries of children, with females being more susceptible to dental caries (Veerasamy et al. 2016), whilst in other studies, boys exhibited higher dental caries experience than girls of the same age (Cagetti et al. 2016). Meanwhile, there have been studies which depict no, or insignificant, sex inequalities in caries experience amongst adolescents (Elias-Boneta et al. 2016). In relation to periodontal (gum) disease, some studies reported that male children had poorer oral hygiene behaviours than females, leading to higher prevalence of periodontal disease and gingivitis (Elias-Boneta et al. 2018). Other studies did not find evidence of sex inequalities in the periodontal status amongst children (Kissa et al. 2016).

Effective oral hygiene behaviours and a healthy diet can potentially mediate the association between SES and oral health for adults (Pang et al. 2022). Additionally, sex can also influence oral health through comorbidities, attitudes, behaviours, and perceptions about oral health in the United States of America (Su et al. 2022). Whilst the mediating effects of health behaviours on the relationship between both sex and SES and oral health have been studied in adults (Su et al. 2022; Pang et al. 2022; Guarnizo-Herreño et al. 2021), there is less research focussing on these mediating factors in children. The National (England, Wales, and Northern Ireland) dental health survey in children (National statistics 2015) provides an opportunity to examine children and adolescents’ oral health inequalities, where oral disease in children is preventable yet prevalent, to address public concerns of health inequalities.

**Fig. 1:**
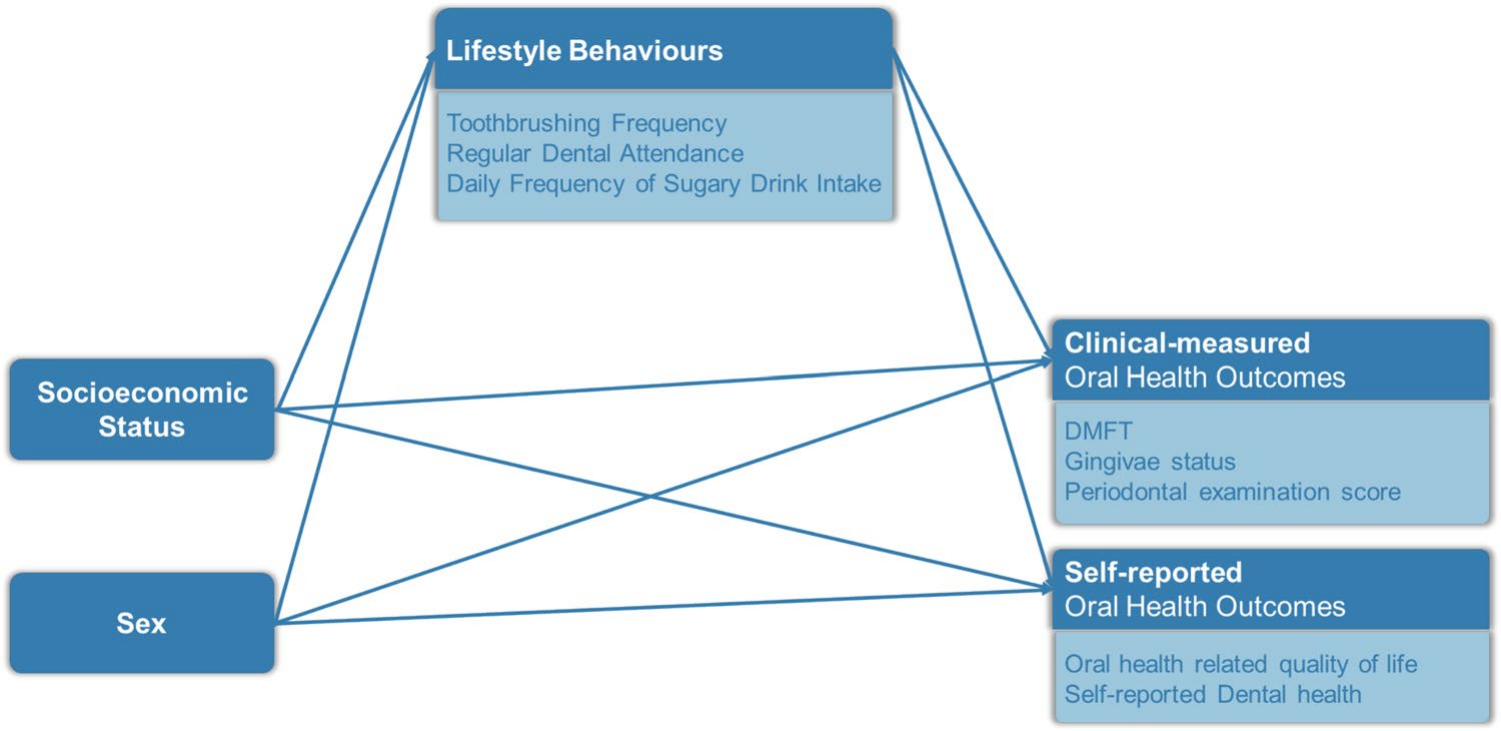
The mediation models of the association between socio-economic status (SES)/sex and oral health outcomes. Arrows depict hypothesised pathways from SES or sex (exposure) to oral health outcome (clinical measured: DMFT, gingival status, periodontal examination score; or self-reported: dental health, oral health-related quality of life) operating via intermediate behavioural factors (tooth brushing frequency, dental attendance pattern, and sugary drink consumption). A direct pathway from SES to the oral health outcome is also shown, representing effects not explained by the measured mediators

The aims of the present study are to: (1) explore sex and SES inequalities in dental caries experience, self-reported oral health, and periodontal status using the Children’s Dental Health Survey 2013 for England, Wales, and Northern Ireland (CDHS 2013), three of the four nations of the United Kingdom (National statistics 2015); and (2) investigate whether children’s oral health inequality can be mediated by lifestyle behaviours (Fig. 1).

## Material and methods

This study followed the STROBE guidelines for cross-sectional studies (von Elm et al. 2007) and involved secondary analysis of national data.

### Data source

The CDHS 2013 dataset included children from England, Wales, and Northern Ireland (National statistics 2015). Data were collected through clinical examinations by calibrated dental teams. Questionnaires were completed by 12- and 15-year-old pupils and parents of 5- and 8-year-old participants. The target groups were 5-, 8-, 12-, and 15-year-old children, representing primary (5 years old), mixed (8 years old), and permanent dentitions (12 and 15 years old).

The CDHS 2013 collected participants’ demographic background information including age, sex (male, female), ethnicity (white, other), and deprivation index as an indicator for social economic status (SES). SES is categorised into three groups: more deprived (HMRC deprivation ranks 1—6496)), medium deprived (ranks 6497—19,489), and less deprived (ranks 19,490–32,482) (Hong et al. 2018).

Ethical approval was gained from the University ethics committee at University College London (Project ID 2000/003) and participants had consented in advance for use of data in future research projects. The CDHS 2013 data are publicly accessible and free to download to all registered users in the UK Data Service (10.5255/UKDA-SN-7774-1) (National statistics 2015); hence, no further ethical approval was required to analyse the data.

### Outcome measures

There were five oral health outcome measures utilised within the data:

1. Dental caries experience based on decayed, missing, and filled teeth (DMFT for permanent and dmft for primary dentition scores, scale variable, range 0—28). Caries experience was measured at two different thresholds in CDHS 2013: (1) enamel caries (lesion confined to enamel but not into dentine); (2) dentine caries (cavitated lesion extending into dentine). This study focussed on dentine lesions only, and DMFT/dmft score are based on dentine-level caries. This applies to all age group.
2. Oral health-related quality of life (OHRQoL): The question asking, “In the last three months have you had difficulty eating/speaking clearly/cleaning your teeth/relaxing (including sleeping)/felt different/smiling, laughing and showing your teeth without being embarrassed/doing your school work/enjoying being with people because of problems with your teeth and mouth?”. This 8 item OHRQoL is recorded as 0 if none of those difficulties was selected, and 1 if else. This measure applies to only 12 and 15 years old, and is known as Children’s version of Oral Impacts on Daily Performances (Child-OIDP, 8-itemised measures adapted from adult 8-itemised OIDP, and validated by many studies) (Gherunpong et al. 2004; Yusuf et al. 2006; Mathur et al. 2022).
3. Self-reported dental health (SRDH): The question “Overall, would you say that your dental health (that is the health of your teeth and mouth) is…”, has five selections (Very good, good, fair, poor, or very poor). Self- reported dental health is coded 0 when participants choose ‘Very good’ or ‘good, or 1 when it is reported as ‘fair’, ‘poor’, or ‘very poor’(Csikar et al. 2016; Mohd Khairuddin et al. 2025). Only 12 and 15 years old reported this information.
4. Gingival status was examined by dental clinicians and was recorded as ‘healthy’ (no treatment is needed) or ‘not healthy’. Only 12 and 15 years old have taken the examination.
5. Periodontal status was clinically examined using basic periodontal examination (BPE) scores for 15-year-old children only (Cole et al. 2014): BPE = 0 as no bleeding; 1 = bleeding on probing; 2 = bleeding with retentive factors such as calculus; 3 = bleeding with shallow pockets between 3.4 and 5.5 mm; and 4 = deep pockets > 5.5 mm.

### Potential mediating factors

The selected potential mediating factors for sex and SES’ association with oral health outcomes were based on evidence from the existing literature and the availability of the CDHS data (Hong et al. 2018; Mohd Khairuddin et al. 2024; Gupta et al. 2021; Office for Health Improvement and Disparities 2021). If the proportion of missing data was higher than 30%, this mediator would not be considered in the analysis (Schulz and Grimes 2002). Mediation analysis was performed for the 12- and 15-year-old cohorts, because the mediators were only available in those age groups, adolescents having self-reported through completion of a questionnaire. Mediation effect was assessed for the association of three potential mediating factors (one at a time) on the sex and SES difference in the risk of poor oral health. The three potential mediating factors include tooth brushing frequency (twice or more, less than twice-a-day), regular dental checkups (regular checkup, or only when having trouble/never) (Mohd Khairuddin et al. 2024), and daily frequency of sugary drink intake (more than 4 times, 3 times, or less) (Hong et al. 2018).

For children’s dental caries, potential mediators include fluoride use, such as fluoride-containing toothpaste, fluoridated vanish, and water fluoridation in specific geographical areas (Iheozor-Ejiofor et al. 2024). However, in the CDHS 2013 dataset, only the use of fluoride drops or tablets in the previous year was recorded. Due to the high percentage of missing data for this variable (76.05%), factors related to fluoride were excluded from the analysis. However, in the UK since the 1970s, the proportion of toothpaste sold which contained fluoride increased from 5% in the 1970s to 96% in 1980s, and nowadays, almost all family toothpastes contained fluoride (Gupta et al. 2021; Marinho 2009). Therefore, toothbrushing could be considered a proxy for fluoride application as well as plaque removal in this study.

### Statistical analysis

Descriptive statistics was performed for all variables stratified by sex and SES. Mean and standard deviation (SD) were reported for continuous data, and frequency (%) for categorical data. Data were weighed according to primary sampling unit and strata. Participants with missing data were excluded in the descriptive table.

Negative binomial models were used to fit the DMFT/dmft scores, and logistic regression models were used for binary oral health outcomes. This study focussed on the identification of factors that mediate the association of sex and SES with oral health outcomes, including DMFTs, SRDH, OHRQoL, gingivae status, and BPE. This study used the difference in coefficients which is the change in the logistic regression or negative binomial model’s beta coefficient associated with sex or SES (adjusted for the other covariates), and then assessed with subsequent adjustment for potential mediating factors. The mediation analyses were performed for 12- and 15-year-old cohorts only.

Mediation analyses were performed using the following steps: 1) the total effects of SES and sex on each oral health outcome, and it was not necessary to observe a statistically significant relationship between sex or SES with oral health outcomes for the further mediation analyses, because no correlation does not disprove causation (Bollen and Noble 2011); 2) sex and SES effect on the mediator, and if the effect is not statistically significant, there was no point for mediation analysis, 3) mediator’s effect on oral health outcomes. As all participants in each group were of the same age, whilst SES and sex were defined as the exposures, no adjustment for demographics was necessary. No other potential confounders were recorded in the dataset, nor were any such factors identified a priori. Average causal mediation effect (ACME) and average direct effect (ADE), and proportion of the effect of sex and SES on the oral health outcomes that go through the mediator were calculated using 100 bootstrapped samples, and 95% confidence interval was computed by determine the indirect effects at the 2.5 and 97.5 percentiles.

The statistical significance level was set at 0.05. Statistical analysis was performed using R Studio with various packages including Survey, mediation, pscl, MASS, and other Packages (R-Studio Team 2025). Complete mediation is defined when ADE is not statistically significant and ACME is statistically significant, indicating the initial effect of exposure on outcome disappears when mediator is included in the model. Incomplete mediation or partial mediation occurs when both ACME and ADE are statistically significant, indicating that the mediator explains a significant part of the relationship between exposure and outcome, but there is still a residual direct effect not explained by the mediator. (MacKinnon and Luecken 2011).

## Results

In total, 9,866 children were included in the present study, of whom 5,054 (51.2%) were female. Regarding SES, 3,101 (31.4%) participants were from more deprived SES background, whilst 4,061 (41.2%) were from medium deprived and 2,302 (23.3%) from less deprived SES. Amongst 12 and 15 years old who completed dental questionnaires regarding their oral hygiene behaviours and lifestyle, females reported better oral hygiene routines than males (14.3% vs 29.1% who brushed less than twice-a-day, *p* < 0.001). However, the proportion of females reporting good dental health was lower than that of males (24.1% in females vs 35.8% in males, *p* < 0.001). The average DMFT score was also higher in females (1.1(SD 1.9) vs 0.8 (SD 1.7), p = 0.027). Besides, periodontal status (measured by BPE score) varied by SES: children from higher SES backgrounds were more likely to have healthy gums (49.1%, 56.4%, and 58.4% across increasing SES groups, p = 0.019), whilst the proportion with a BPE score of 1 decreased with increasing SES (57.9%, 46.7%, and 41.0%, p = 0.023).

Disparities in oral hygiene behaviours and oral health (dental caries experience, self-reported oral health, BPE, and gingival status) were also evident across SES categories. The proportion of participants reporting brushing their teeth less than twice-a-day was higher in the more deprived group compared with the less deprived group (26.1% vs 16.2%, respectively, *p* < 0.001, Table 1). Likewise, participants from more deprived areas were more likely to report visiting a dentist only when experiencing problems (29.5% in more deprived groups vs 7.9% in less deprived groups).

**Table 1:** Descriptive table on participants in 2013 CDHS, by sex and social economic status (SES), weighted by survey weight and strata. *N* = 9866

	Sex			SES			
	Male	Female	*P*	More deprived	Medium deprived	Less deprived	*P*
n	4812	5054		3101	4061	2302	
**Demography**							
Age (%)			0.954				0.847
5 years old	1264 (27.3)	1285 (27.0)		749 (27.8)	1079 (27.5)	622 (26.2)	
8 years old	1171 (24.4)	1196 (24.6)		660 (23.6)	996 (24.5)	616 (25.4)	
12 years old	1222 (24.3)	1310 (24.0)		890 (25.2)	989 (23.4)	551 (24.2)	
15 years old	1155 (24.0)	1263 (24.4)		802 (23.4)	997 (24.7)	513 (24.2)	
Sex, Female (%)				1669 (51.4)	2047 (49.9)	1119 (47.3)	0.273
Ethnicity, others (%)	728 (20.2)	764 (20.2)	0.952	769 (34.4)	515 (17.8)	154 (7.8)	< 0.001
SES (%)			0.273				< 0.001
more deprived	1432 (30.3)	1669 (32.5)					
medium deprived	2014 (40.3)	2047 (40.7)					
less deprived	1183 (29.4)	1119 (26.8)					
**Oral hygiene behaviours**							
Daily toothbrushing frequency, less than twice a day (%)	719 (29.1)	385 (14.3)	< 0.001	436 (26.1)	450 (22.7)	180 (16.2)	< 0.001
Dental checkups, Only when in trouble/never (%)	428 (19.5)	432 (16.9)	0.33	440 (29.5)	301 (16.8)	80 (7.9)	< 0.001
Daily sugary drink intake frequency, Less than four times a day (%)	1501 (67.8)	1673 (69.6)	0.379	915 (58.3)	1332 (70.7)	792 (77.2)	< 0.001
**Oral health outcome**							
Gingival status, healthy gums (%)	2576 (54.3)	2777 (54.9)	0.732	1469 (49.1)	2305 (56.4)	1374 (58.4)	0.019
dmfts (mean (SD))	0.8 (1.7)	0.7 (1.6)	0.056	0.8 (1.9)	0.8 (1.8)	0.5 (1.3)	< 0.001
DMFTs (mean (SD))	0.8 (1.7)	1.1 (1.9)	0.027	1.0 (2.0)	0.8 (1.9)	0.4 (1.3)	< 0.001
OHRQoL, with difficulty (%)	1175 (23.5)	1437 (26.7)	0.121	947 (26.7)	1052 (25.8)	493 (21.7)	0.084
SROH, fair or worse (%)	846 (35.8)	630 (24.1)	< 0.001	564 (34.6)	606 (32.3)	250 (22.5)	< 0.001
BPE, 1 (%)	577 (48.7)	622 (49.4)	0.889	459 (57.9)	458 (46.7)	222 (41.0)	0.023

SD, standard deviation; N, number of participants

Data are presented as frequency (%) unless specified. Numbers may not sum to totals due to missing values; percentage may not sum to 100 due to rounding. Variables with missing data: HMRC quintile (402, 4.1%). Note that oral hygiene habits and dietary habits were from questionnaires only responded by 12 and 15 years old. BPE was available in the 15-year group only

Deprivation index was grouped in quintiles as follows:

1 (most deprived, ranks 1–6496), 2 (ranks 6497–12,993), 3 (ranks 12,994–19,489), 4 (ranks 19,490–25986), 5 (least deprived, ranks 25,987–32,482)

*P* value was for comparing characteristics between male and female, or amongst the three categories of SES

### Five- and eight-year-old groups

The only dental assessment for 5- and 8-year-old groups was the dmft score, and 5 years old had a mean dmft of 1.2 (SD 2.3), whilst the dmft for 8 years old was 1.5 (SD 2.1). Sex was not associated with variation in dmft, but higher SES was associated with a less dental caries experience, especially in the younger group (Incident rate ratio, IRR: 0.69, 95% confidence interval CI: 0.62—0.77 amongst 5 years old; IRR 0.84, 95% CI: 0.77—0.91 amongst 8 years old).

### Twelve- and fifteen-year-old groups

The oral health disparity due to sex and SES inequalities begins to show amongst the 12- and 15-year-old groups; apart from the lack of evidence showing direct effect of sex on OHRQoL in 12-year-old group and BPE in 15-year-old group, every other oral health outcome was found to be directly affected by sex and SES, for both 12- and 15-year-old groups (Tables 2 and 3).

**Table 2:** Potential mediators and its mediation effect on oral health (DMFT, oral health-related quality of life, self-reported dental health and gingival status) amongst **12 years olds**

Oral health outcomes	Mediator	Sample size	Exposure—> mediator	Mediator− > OHO	Exposure—> OHO (total effect)	ACME (95% CI)	ADE (95% CI)	Proportion Mediated	Conclusion
**SES to OH Outcomes**									
DMFTs	Toothbrushing frequency	2532	−0.29***	0.32***	−0.41***	−0.03 (−0.05, −0.01)***	−0.55 (−0.67, −0.42)***	0.05 (0.01, 0.09)***	Incomplete mediation effect
	Regular dental attendance	2532	−0.82***	0.44***	−0.41***	−0.10 (−0.12, −0.06)***	−0.561 (−0.65, −0.38)***	0.16 (0.10, 0.20)***	Incomplete mediation effect
	Sugary drink intake frequency	2532	−0.49***	0.28***	−0.41***	−0.05 (−0.07, −0.02)***	−0.54 (−0.67, −0.37)***	0.08 (0.04, 0.12)***	Incomplete mediation effect
OHQoL (0 as no impact, 1 as has impact)	Toothbrushing frequency	2532	−0.29***	0.49***	−0.12*	−0.002 (−0.011, 0.00)***	−0.02 (−0.05, −0.01)***	0.08(0.07, 0.55)***	Incomplete mediation effect
	Regular dental attendance	2532	−0.82***	0.51***	−0.12*	−0.017 (−0.023, −0.01)***	−0.016 (−0.041, 0.01)	0.51 (0.22, 1.93)***	Complete mediation effect
	Sugary drink intake frequency	2532	−0.49***	0.29**	−0.12*	−0.01 (−0.02, 0.00)***	−0.02(−0.04, 0.01)	0.28(−1.4, 1.28)	Complete mediation effect
SRDH (0 good, 1 bad)	Toothbrushing frequency	2532	−0.29***	1.06***	−0.19**	0.0002 (−0.002, 0.00)	−0.03 (−0.05, −0.01)	−0.007(−0.07, 0.15)	No mediation effect
	Regular dental attendance	2375	−0.83***	1.09***	−0.19**	−0.017(−0.023, −0.01)***	−0.01 (−0.03, 0.01)	0.76 (0.49, 1.70)***	Complete mediation effect
	Sugary drink intake frequency	2532	−0.49***	0.29**	−0.19**	−0.01 (−0.01, 0.00)***	−0.02(−0.05, 0.01)	0.25 (−0.75, 0.80)	Complete mediation effect
Gingivae status (0 good, 1 bad)	Toothbrushing frequency	2532	−0.29***	0.62***	−0.25***	−0.005 (−0.01, 0.00)***	−0.05 (−0.07, −0.03)***	0.09(0.05, 0.25)***	Incomplete mediation effect
	Regular dental attendance	2532	−0.82***	0.28*	−0.25***	−0.007 (−0.02, 0.00)*	−0.05 (−0.07, −0.03)***	0.13 (0.03, 0.31)*	Incomplete mediation effect
	Sugary drink intake frequency	2532	−0.49***	0.33**	−0.25***	−0.01 (−0.02, 0.00)***	−0.05 (−0.08, −0.03)***	0.17 (0.06, 0.25)***	Incomplete mediation effect
**Sex to OH outcomes**									
DMFTs (scale, 0–28)	Toothbrushing frequency	2532	0.83***	0.32***	−0.22**	0.06 (0.04, 0.11)***	−0.34 (−0.49, −0.12)***	−0.21 (−0.68, −0.10)*	Incomplete mediation effect
OHQoL (0 as no impact, 1 as has impact)	Toothbrushing frequency	2532	0.83***	0.49***	−0.08	0.02 (−0.01, 0.03)***	−0.04 (−0.07, −0.01)*	−0.86 (−9.47, 5.17)	Complete mediation effect
SRDH (0 good, 1 bad)	Toothbrushing frequency	2532	0.83***	1.06***	0.54***	−0.03 (−0.04, −0.02)***	0.01 (−0.03, 0.05)	1.55 (−14.27, 14.16)	Complete mediation effect

SES, socio-economic status; OHO, oral health outcome; ACME, average causal mediation effect; ADE, average direct effect; CI, confidence interval; DMFT, decayed, missing and filled teeth; OHRQoL, Oral health-related quality of life; SRDH, Self-reported dental health

^*^p value < 0.05

^**^p value < 0.01

^***^p value < 0.001

**Table 3:** Potential mediators and its mediation effect on oral health (DMFT, oral health-related quality of life, self-reported dental health, gingival status, and BPE) amongst **15 years olds**

Oral health outcome	Mediator	Sample size	Exposure—> mediator	Mediator- > OHO	Exposure—> OHO (total effect)	ACME (95% CI)	ADE (95% CI)	Proportion mediated	Conclusion
**SES to OHO**									
DMFTs	Toothbrushing frequency	2418	−0.37***	0.18*	−0.29***	−0.03 (−0.05, 0.00)*	−0.71 (−0.95, −0.57)***	0.04 (0.01, 0.07)*	incomplete mediation effect
	Regular dental attendance	2418	−0.65***	0.39***	−0.29***	−0.1 1(−0.16, −0.07)***	−0.6 5(−0.89, −0.46)***	0.14 (0.10, 0.22)***	incomplete mediation effect
	Sugary drink intake frequency	2418	−0.49***	0.36***	−0.29***	−0.12 (−0.14, −0.05)***	−0.64 (−0.81, −0.44)***	0.15(0.08, 0.19)***	incomplete mediation effect
OHQoL (0 as no impact, 1 as has impact)	Toothbrushing frequency	2418	−0.37***	0.42***	−0.23***	−0.001 (−0.01, 0.00)***	−0.05 (−0.08, −0.03)***	0.12 (0.03, 0.26)***	incomplete mediation effect
	Regular dental attendance	2418	−0.65***	0.56***	−0.23***	−0.01 (−0.02, −0.01)***	−0.05 (−0.07,− 0.02)***	0.22 (0.12, 0.54)***	incomplete mediation effect
	Sugary drink intake frequency	2418	−0.49***	0.44**	−0.23***	−0.01 (−0.02, −0.01)***	−0.04 (−0.07, −0.02)***	0.19 (0.11, 0.43)***	incomplete mediation effect
SRDH (0 good, 1 bad)	Toothbrushing frequency	2262	−0.36***	1.10***	−0.37**	−0.01 (−0.02, −0.01)***	−0.06 (−0.09, −0.03)***	0.18 (0.10, 0.34)***	incomplete mediation effect
	Regular dental attendance	2375	−0.65***	1.08***	−0.37**	−0.03 (−0.04, −0.02)***	−0.0 5(−0.07, −0.03)***	0.34 (0.21, 0.53)***	incomplete mediation effect
	Sugary drink intake frequency	2227	−0.50***	0.37**	−0.37**	−0.01 (−0.01, 0.00)***	−0.07 (−0.10, −0.04)***	0.12 (0.05, 0.22)	incomplete mediation effect
Gingivae status (0 good, 1 bad)	Toothbrushing frequency	2418	−0.37***	0.61***	−0.37**	−0.01 (−0.02, 0.00)***	−0.08 (−0.11, −0.06)***	0.09 (0.05, 0.17)***	incomplete mediation effect
	Regular dental attendance	2418	−0.65***	0.59***	−0.37**	−0.02 (−0.02, −0.01)***	−0.08 (−0.11, −0.06)***	0.18 (0.09, 0.24)***	incomplete mediation effect
	Sugary drink intake frequency	2418	−0.49***	0.41**	−0.37**	−0.01 (−0.02, 0.00)***	−0.08 (−0.11, −0.06)***	0.13 (0.06, 0.20)***	incomplete mediation effect
BPE (0 good, 1 bad)	Toothbrushing frequency	2172	−0.37***	0.53***	−0.32***	−0.01 (−0.02, 0.00)***	−0.07 (−0.10, −0.04)***	0.10 (0.04, 0.20)***	incomplete mediation effect
	Regular dental attendance	2176	−0.66***	0.61***	−0.32***	−0.01 (−0.02, −0.01)***	−0.06 (−0.10, −0.04)***	0.17 (0.09, 0.32)***	incomplete mediation effect
	Sugary drink intake frequency	2139	−0.10***	0.38**	−0.32***	−0.01 (−0.02, −0.01)***	−0.07 (−0.10, −0.04)***	0.12 (0.06, 0.23)***	incomplete mediation effect
**Sex to OHO**									
DMFTs	Toothbrushing frequency	2418	1.12***	0.18*	−0.18**	0.07 (0.03, 0.13)***	−0.48 (−0.72, −0.22)***	−0.18(−0.58, −0.06)*	incomplete mediation effect
OHQoL (0 as no impact, 1 as has impact)	Toothbrushing frequency	2418	1.12***	0.42***	−0.36***	0.02 (0.01, 0.03)***	−0.10 (−0.14, −0.07)***	−0.19 (−−0.51,−0.11)***	incomplete mediation effect
SRDH (0 good, 1 bad)	Toothbrushing frequency	2418	1.13***	1.10***	0.64***	0.04 (0.03, 0.06)***	0.08 (0.04, 0.12)***	0.34 (0.25,0.51)***	incomplete mediation effect
BPE (0 good, 1 bad)	Toothbrushing frequency	2172	1.14***	0.53***	0.07	0.02 (0.01, 0.03)***	−0.01 (−0.04, 0.03)	1.39 (−16.46, 66.97)	complete mediation effect

SES, socio-economic status; OHO, oral health outcome; ACME, average causal mediation effect; ADE, average direct effect; CI, confidence interval; DMFT, decayed, missing and filled teeth; OHRQoL, Oral health-related quality of life; SRDH, Self-reported dental health

^*^p value < 0.05

^**^p value < 0.01

^***^p value < 0.001

### Mediation analyses

Amongst 12 years old, regular dental attendance and sugary drink intake largely mediated the effect of SES on children’s SRDH and OHRQoL; toothbrushing frequency largely mediated sex’s impact on SRDH and OHRQoL (Table 2). Amongst 15 years old, toothbrushing frequency largely mediated the impact of sex on BPE (Table 3). Other diet or oral health behaviours all partially mediated the effect of SES or sex on oral health outcomes, except toothbrushing which had no mediation effect on SES to SRDH for 12 years old (Tables 2 and 3).

Amongst 12 years old, 51% (95% CI: 0.22—1.93) of total effect of SES on OHRQoL may be explained by regular dental attendance, whilst the proportion mediated was lower 22% (95% CI: 0.12—0.54) amongst 15 years old. Additionally, amongst 15 years old, toothbrushing frequency mediated 34% (95% CI: 0.25—0.51) of the impact of sex on self-reported oral health.

## Discussion

The association between SES, sex, and oral health amongst UK children was analysed in the present study, demonstrating socio-economic inequalities in children's oral health and a higher susceptibility of female adolescents to dental caries. The present findings suggest that a healthier lifestyle, characterised by toothbrushing frequency twice or more per day, regular dental attendance, and a healthier diet involving less frequent sugary drink consumption, may reduce the inequality of sex and oral health and SES and oral health amongst children. These behaviours are in line with contemporary evidence-based guidance (Office for Health Improvement and Disparities 2021).

### Sex and children’s oral health inequalities

In relation to the impact of inequalities on oral health by sex amongst adolescents, the present findings provide further evidence in this field; it was found that girls were more susceptible to dental caries, whilst sex had little influence on periodontal disease. It is unclear what influences led to these findings for dental caries; it could be associated with earlier tooth eruption and hormonal fluctuations in girls (Hill et al. 2023). Females experience earlier eruption of teeth than males, so their teeth can be exposed longer to a cariogenic oral environment; moreover, the experience of puberty and menstruation may potentially transform the oral environment towards being more cariogenic during this time by modifying the biochemical composition of saliva and overall salivary flow rate (Lukacs and Largaespada 2006). The cumulative effect of these individual factors could be responsible for females presenting with more dental caries and would benefit from further research.

The proportion mediated by toothbrushing frequency in the association between sex and self-reported dental health was counter-intuitive (females who had a higher toothbrushing frequency are more likely to self-rate as having bad dental health), potentially indicating a suppressed effect of sex through toothbrushing on self-reported dental health. This may reflect the paradox that, although females are more likely to brush twice daily, they also tend to report greater anxiety and lower self-rated oral health which may be related to their self-perceptions (Ostberg et al. 1999).

### Socio-economic status and children’s oral health inequalities

Consistent with previous studies, the results of oral health inequalities amongst different SES in children are not surprising (Office for Health Improvement and Disparities 2021). In the present epidemiological study, it was found that UK children (excluding Scotland) from more deprived areas are more susceptible to dental caries and periodontal disease, which may occur for a wide range of reasons (Nayee et al. 2018; Arora et al. 2021).

#### Toothbrushing (and fluoride application) as mediator

Parents and guardians with lower SES may not prioritise oral health (Nayee et al. 2018)—they may be unaware of the importance of supervised brushing and the benefits of toothbrushing with a fluoride toothpaste for their children, treating oral health not a priority (Marshman et al. 2016; Gray-Burrows et al. 2023). This may result in inadequate and infrequent toothbrushing with an age-appropriate fluoride toothpaste amongst children, consequently increasing the risk of oral diseases. This hypothesis is also supported by the observed negative correlation between toothbrushing frequency and SES in the present study.

#### Diet–sugar intake as mediator

Dietary intake is influenced by the social context (Shaw et al. 2023; Gautam et al. 2023) and consumption of sugary drinks exceeding national recommendation level is a key mediator (Public Health England 2015). In the UK, children consume substantially more free sugar than recommended, with average intakes accounting for 10.5% of total energy, which is more than twice the UK government’s recommended threshold of 5% (Nutrition 2015). Poor dietary habits, especially high consumption of sugar-sweetened beverages, are more prevalent in deprived areas (Mohd Khairuddin et al. 2024).

#### Dental attendance as mediator

The present findings suggest that regular dental attendance is not only crucial for maintaining good oral health but also mediates the impact of SES on all oral health outcomes assessed. Regular dental attendance has a positive impact on oral health, because it ensures continued access to preventive dental care and early intervention for oral health problems, as promoted by national guidance (National Institute for Health and Care Excellence 2015; Office for Health Improvement and Disparities 2021). Whilst across the nations of the UK, economic status theoretically has minimal influence due to the free dental services being provided to children through the NHS, attendance patterns differ by SES, other barriers exist. The present results align with the previous findings that children of higher SES are more likely to receive dental checkups regularly (Mohd Khairuddin et al. 2024). Thus, we speculate that the gap in oral health prioritisation, awareness, and behaviours amongst parents/guardians plays a pivotal role in this mediating process—advantaged families may exhibit favourable NHS utilisation frequency and patterns and seek additional preventive dental service, whilst disadvantaged families may only tend to attend services symptomatically. Besides, regular dental checkups can allow primary and secondary prevention of disease, pain and discomfort, and could make children feel more confident about their oral health (National Institute for Health and Care Excellence 2015; Office for Health Improvement and Disparities 2021). These effects could apply to children regardless of SES, thus moderating the effects of SES on both self-perceived and practical oral health. Most children attend a dentist regularly and when they do, this presents an important opportunity to affirm positive health behaviours, and promote behaviour change to address common risk factors relating to hygiene, fluoride, and diet (Public Health England 2021) Additionally, the necessity of general children’s dental preventive services (National Institue for Health and Care Excellence 2015) is often not utilised. Although across the three nations of the UK involved in this study, dental care delivered by the NHS is free at the point of delivery (ideally providing regular dental checkups, together with caries preventive treatments, such as fluoride varnish, fissure sealants, and dental health education in line with the contemporary evidence base), children from families of low SES are less motivated to access care regularly, despite potentially having higher levels of oral disease (Office for Health Improvement and Disparities 2021).

These factors exacerbate the adverse impact of low SES on oral health. Taken together, these findings highlight a structural problem in the means of acquiring and maintaining oral health and nutrition behaviours, which limits the capacity of families from lower SES backgrounds to establish and maintain healthy behaviours.

It is important to acknowledge that increased knowledge about the importance of oral health and the necessity of oral hygiene practices does not necessarily directly translate into behaviour change (Peerbhay et al. 2025). Therefore, behavioural, community, and societal interventions also play a crucial role in improving oral health amongst children (Office for Health Improvement and Disparities 2021; National Institute for Health and Care Excellence 2015). Emerging evidence suggests that oral hygiene behaviour change practices can be implemented at a low cost through simple techniques, such as mobile phone-based interventions. For instance, short message service (SMS) reminders have been proved that it could increase health care appointment attendance and toothbrushing frequency in adolescents (Innes et al. 2024). Scotland, however, through its Childsmile programme has achieved a reduction in oral health inequalities through targeted toothbrushing with a fluoride toothpaste for children in early years through community initiatives to reach families which may not access dental care service regularly which provides a better start in life (Bauld 2025).

### Strengths and limitations

The present study has several strengths. In the view of the authors, this is the first study to investigate the mediation effect of oral health behaviours to understand the oral health inequality in the children from England, Wales and Northern Ireland. It, thus, provides evidence regarding the mechanisms through which SES and sex impact oral health via oral health behaviours, and demonstrates the importance of regular dental visit, maintaining good oral hygiene, and consumption of fewer sugary beverages in line with contemporary guidance (Office for Health Improvement and Disparities 2021). A large, representative database and robust analyses on five oral health measurements was used to comprehensively investigate the impact of healthier behaviours to tackle health inequality issues in the UK.

Despite the strengths, there are limitations which should be acknowledged. First, this study is cross-sectional rather than longitudinal, which limited the analysis to associations rather than causal relationships between SES, sex, and oral health. Second, the health behaviours analysed are self-reported by adolescents, which may introduce reporting bias and limit the accuracy of the findings (Goodwin et al. 2025). Third, uncaptured confounding factors may also contribute. These results should therefore be interpreted with caution, and further research is needed to confirm whether these negative mediation estimates represent genuine mechanisms. Moreover, the measurement methods of periodontal status can be limited by the use of the BPE index and binary gingival status. These methods serve as proxies of periodontal disease, potentially leading to an over- or under-estimation of its true prevalence. However, as periodontal disease is relatively uncommon amongst children, these measures are considered acceptable for population-based surveillance. Toothbrushing frequency was used as a proxy for fluoride application due to missing data. However, this measure does not capture all sources of fluoride, including professional applications such as fluoride varnishes during dental visits, but it represents a common route of exposure outside of community water fluoridation in the UK. As a result, brushing frequency alone may not fully reflect fluoride exposure, and the effect of fluoride could be underestimated or misclassified in this study.

In conclusion, the key findings were: (1) child oral health inequality existed by sex and socio-economic status; (2) healthier lifestyle behaviours can mediate the effect of sex and SES on children’s oral health which, thus, reduce oral health inequality. Therefore, we urge that oral health providers deliver and promote evidence-based preventive care and healthier lifestyle behaviours amongst young children and adolescents as well as working at community and policy levels to tackle inequalities. Paediatric dentists can play a major leadership role in influencing all members of the dental team in supporting their patients in preventive care including risk-based prevention to support the most high-risk children. The findings suggest that by incorporating simple daily steps, such as (at least) twice daily toothbrushing with a fluoride toothpaste, drinking less sugary beverages, and regular dental attendance, the impact of sex and SES inequality on children`s oral health may be minimised. These steps not only reduce the occurrence of oral diseases in children but also offer long-term health benefits and improve overall quality of life.

## Bullet points

Why this paper is important to paediatric dentists?

- Sex and socio-economic status influence children's oral health inequality.
- The importance of healthier lifestyle which can reduce the disparities of oral health of children of different background is emphasised.
- Optimal health behaviours, such as toothbrushing frequency ≥ twice daily, less sugary drink consumption, and regular dental visit, could reduce oral health inequalities dramatically.

## Data Availability

The CDHS 2013 data is publicly accessible and free to download to all registered users in the UK Data Service (10.5255/UKDA-SN-7774-1) (National statistics 2015); hence, no further ethical approval was required to analyse the data.
